# Supplementary material for: Regional mechanical dyssynchrony and shortened systole are present in people with Takotsubo syndrome
Source: Commun Med (Lond). 2024 Nov 1;4:223. doi: 10.1038/s43856-024-00641-5 (PMC11530451; doi:10.1038/s43856-024-00641-5)
Supplement: Supplementary file 2 — Supplementary Information [file 43856_2024_641_MOESM2_ESM.pdf]

Supplementary Information

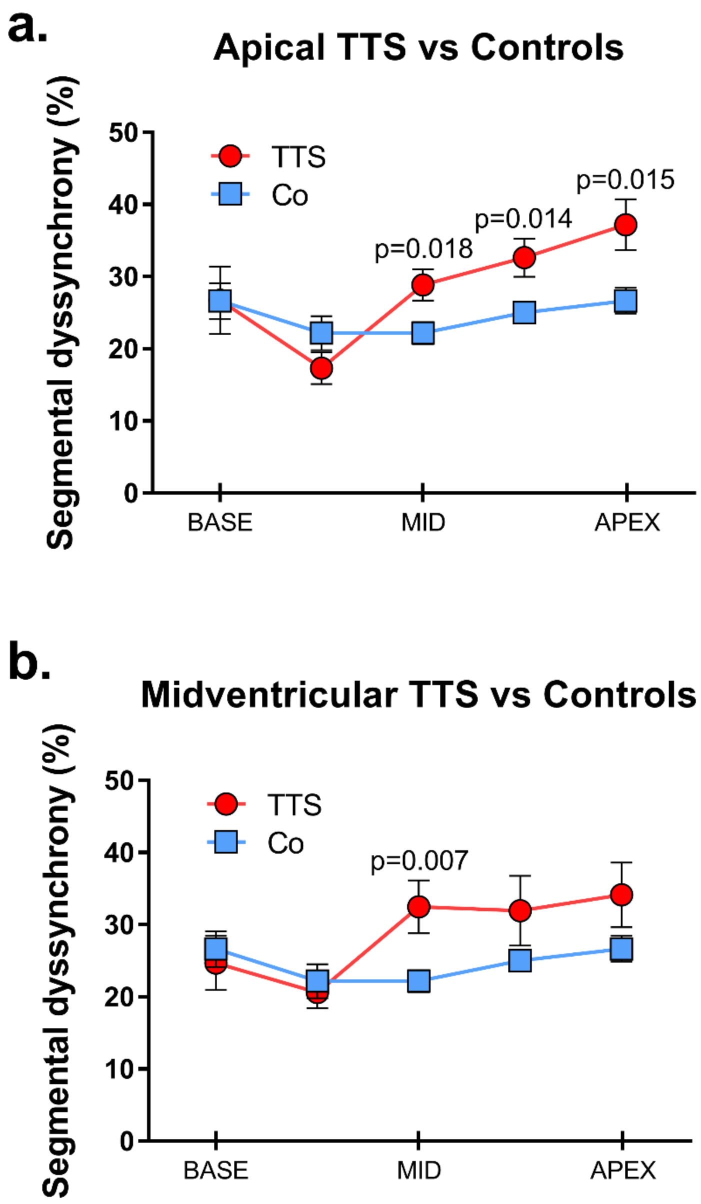

Supplemental Figure. Segmental Dyssynchrony in Takotsubo Syndrome. (a) Segmental dyssynchrony comparison between apical Takotsubo syndrome (TTS) patients and controls (Co, n=14). The graph illustrates the percentage of segmental dyssynchrony in apical TTS patients (n=14) and controls across different heart segments: base (5 to 4), mid (3), and apex (2 to 1). Apical TTS patients exhibit significantly higher segmental dyssynchrony in the mid and apical segments compared to controls. The overall segmental dyssynchrony in apical TTS patients shows a notable increase from base to apex, suggesting a gradient of mechanical inefficiency progressing towards the apex. Exact p-values are presented, and statistical significance is assumed when $p<0.05$,

compared to controls. Statistical analysis shows a significant difference in dyssynchrony between TTS and controls. (**b**) Segmental dyssynchrony comparison between midventricular TTS patients and controls. The graph displays the percentage of segmental dyssynchrony in midventricular TTS patients (n=8) and controls across the base, mid, and apex segments. Midventricular TTS patients show significantly higher dyssynchrony in the mid segment compared to controls. Although there is an observable trend of increased dyssynchrony towards the apex, the difference is less pronounced compared to apical TTS patients, potentially due to the smaller sample size. Data are presented as mean ± standard error of the mean (SEM).
