# Supplementary material for: Regional mechanical dyssynchrony and shortened systole are present in people with Takotsubo syndrome
Source: Commun Med (Lond). 2024 Nov 1;4:223. doi: 10.1038/s43856-024-00641-5 (PMC11530451; doi:10.1038/s43856-024-00641-5)
Supplement: Supplementary file 3 — Description of Additional Supplementary Files [file 43856_2024_641_MOESM3_ESM.pdf]

Description of Additional Supplementary Files

File name: Supplementary Data 1

File description : contains the data underlying Figure 2.

File name: Supplementary Data 2

File description: contains the data for Figure 4.

File name: Supplementary Data 3

File description: contains the data for Figure 5.

File name: Supplementary Data 4

File description: contains the data for the Supplementary Figure
